# Efficient Recovery Annealing of the Pseudocapacitive Electrode with a High Loading of Cobalt Oxide Nanoparticles for Hybrid Supercapacitor Applications

**DOI:** 10.3390/nano12203669

**Published:** 2022-10-19

**Authors:** Khabibulla A. Abdullin, Maratbek T. Gabdullin, Zhanar K. Kalkozova, Shyryn T. Nurbolat, Mojtaba Mirzaeian

**Affiliations:** 1 National Nanotechnology Laboratory of Open Type (NNLOT), Al-Farabi Kazakh National University, Al-Farabi Avenue 71, Almaty 050012, Kazakhstan; 2 Institute of Applied Science & Information Technology, Shashkin Str. 40–48, Almaty 050040, Kazakhstan; 3 Research Center of Renewable Energy and Nanotechnology, Kazakh-British Technical University, Tole bi st. 59, Almaty 050000, Kazakhstan; 4 School of Computing, Engineering and Physical Sciences, University of the West of Scotland, Paisley PA1 2BE, UK

**Keywords:** supercapacitors, electrode materials, hybrid capacitors, energy storage devices

## Abstract

Electrochemical pseudocapacitors, along with batteries, are the essential components of today’s highly efficient energy storage systems. Cobalt oxide is widely developing for hybrid supercapacitor pseudocapacitance electrode applications due to its wide range of redox reactions, high theoretical capacitance, low cost, and presence of electrical conductivity. In this work, a recovery annealing approach is proposed to modify the electrochemical properties of Co_3_O_4_ pseudocapacitive electrodes. Cyclic voltammetry measurements indicate a predominance of surface-controlled redox reactions as a result of recovery annealing. X-ray diffraction, Raman spectra, and XPES results showed that due to the small size of cobalt oxide particles, low-temperature recovery causes the transformation of the Co_3_O_4_ nanocrystalline phase into the CoO phase. For the same reason, a rapid reverse transformation of CoO into Co_3_O_4_ occurs during in situ oxidation. This recrystallization enhances the electrochemical activity of the surface of nanoparticles, where a high concentration of oxygen vacancies is observed in the resulting Co_3_O_4_ phase. Thus, a simple method of modifying nanocrystalline Co_3_O_4_ electrodes provides much-improved pseudocapacitance characteristics.

## 1. Introduction

The widespread use of electricity in transport, autonomous systems, and various gadgets requires the creation of universal energy storage/supply systems, with a wide range of power density and energy density. Electrochemical energy-storage/power-supply systems consist of energy storage batteries and high-power density supercapacitors. However, in order to fill the gap in energy/power capability of these technologies when combined together to create systems with a continuous energy and power storage/delivery spectrum and fulfill the ever-increasing power demands of newly emerging devices, the evolution of hybrid supercapacitors is crucial and has attracted huge interest in recent years with a large number of studies focusing on the development of hybrid supercapacitors and materials for such device structures published [1,2,3,4]. According to the charge storage mechanism, supercapacitors are classified into three main types: (i) electric double-layer capacitors, (ii) pseudocapacitors, and (iii) hybrid supercapacitors. Electric double layer capacitors with an electrostatic charge storage mechanism most often use carbon nanomaterials as an electroactive material [5]. Pseudocapacitors use the Faraday mechanism of charge accumulation on the surface and in the near-surface layer of electrodes mainly made from transition metal oxides [6,7,8,9]. In theory, their capacitive ability is expected to be much higher than that of the double-layer capacitors. Hybrid supercapacitors typically consist of a Faraday electrode as the energy source and a capacitor-type electrode as the power source. They exhibit a much higher capacitance for pulse power and higher energy densities when compared to previous types. Hybrid supercapacitors have been intensively developing recently, and a number of reviews are devoted to promising materials and device structures for creating hybrid supercapacitors [6,7,8,9,10,11,12,13,14,15].

A wide range of materials and composites are considered suitable for use in hybrid capacitors, including metal oxides and sulfides [16,17,18], their carbon-based composites [5], conductive polymers [10,19,20,21], flexible materials and composites [22,23]. Transition metal oxides, such as oxides of iron, nickel, manganese, ruthenium, etc., are suitable compounds as an electrode material in supercapacitors and Faraday electrodes of hybrid supercapacitors [24,25] because of their high theoretical capacitance compared to carbon electrodes. A number of reviews on the development of transition metal oxide-based electrodes for hybrid supercapacitors have been published in recent years [10,15,16,21,25,26], indicating intensive research in this area.

Among transition metal oxides, cobalt oxide has a relatively low cost, significant electrical conductivity, low toxicity compared to other oxides such as ruthenium oxide, high theoretical capacitance, and two pairs of redox reactions (Co^3+^/Co^4+^ and Co^2+^/Co^3+^):

Co_3_O_4_ + OH^−^ + H_2_O → 3CoOOH + e^−^, (1)

CoOOH + OH^−^ → 4CoO_2_ + H_2_O + e^−^, (2)

which is favorable for creating supercapacitor electrodes [16,27]. Despite the fact that cobalt oxide shows pronounced redox reactions and is more of a battery-type material, this material is being actively investigated for the creation of supercapacitors, and there are a number of publications devoted to the development of supercapacitor electrodes based on cobalt oxide. Different methods such as the development of zero-dimensional, one-dimensional, two-dimensional, and three-dimensional nanostructures have been introduced to synthesize battery-type Co_3_O_4_-based electrodes with high areal capacity and high mass loading; however, these electrodes suffer from relatively low electronic conductivity and huge volume changes [28]. The emerging additive manufacturing techniques using 3D printing methods such as generic 3D printing, direct ink writing, stereolithography, fused deposition modeling, binder jetting, and also three-dimensional printing based on extrusion have also allowed the design of metal oxide-based electrode structures with high areal and volumetric capacitance for supercapacitor applications [29]. To achieve the high pseudocapacitive characteristics of Co_3_O_4_ electrodes, it is necessary to use nanoparticles or thin films of the oxide that have a large specific surface area and exhibit pseudocapacitive properties.

As is known, nanoparticles and thin films of metal oxides exhibit superb pseudocapacitive properties due to Faraday charge transfer reactions on their surface and in near-surface layers [9,30]. Electrodes made of Co_3_O_4_ nanoparticles also degrade less due to changes in the volume of cobalt oxide during cycling. Therefore, many recent studies have been devoted to the application of nanostructured cobalt oxide for pseudocapacitive electrodes. Various synthesis methods such as chemical precipitation, the hydrothermal method, the sol–gel method, photothermal synthesis, etc., are used for the synthesis of pseudocapacitive materials based on Co_3_O_4_ [31,32,33,34,35]. To achieve high electrochemical parameters, Co_3_O_4_ supercapacitor electrodes are made on the basis of films and porous structures [32,33], composites consisting of an electrically conductive matrix with a large specific surface area, in which Co_3_O_4_ nanoparticles are embedded and fixed in the matrix by adhesion forces or a suitable binder [34,35,36,37,38,39,40,41,42,43,44,45,46,47], and also various binary and ternary systems of oxides of cobalt and Mo, Mn, and Cu [38,44,45,46,47]. Doping is used to improve the electrical conductivity of cobalt oxide [48,49]. Although cobalt oxide has a very high theoretical capacitance (3560 F g^−1^), the practically achievable specific capacitance is much lower. High values of specific capacity are achieved only at low mass loads (<1 mg cm^−2^). For example, a specific capacitance of 3480 F g^−1^ has been achieved with a mass loading of 25 micrograms for Co_3_O_4_ nanoparticles deposited on vertically aligned graphene nanosheets in a symmetrical capacitor at an operating voltage of −0.5 to +0.5 V [50]. A very high specific capacity of 978 F g^−1^ at 1 A g^−1^ was achieved at a loading of 6.5 mg cm^−2^ of nano- Co_3_O_4_/graphene composites obtained by the photothermal method [51], which is apparently due to the high degree of photoreduction in graphene oxide and the fragmentation of Co_3_O_4_ nanorods under laser radiation. In [52], hollow Co_3_O_4_ nanoboxes exhibited a specific capacity of 1832.7 F g^−1^ at 1 A g^−1^ and a cycling stability of 85.9% after 5000 cycles at 1 mg loading. The specific capacitance of about 642 F g^−1^ at 1 A g^−1^ was obtained by loading 2.0 mg cm^−2^ for carbon-supported cobalt oxide nanoparticles [53], and the specific capacitance of 423 F g^−1^ at 1 A g^−1^ was obtained by loading 2.0 mg cm^−2^ for three-dimensional porous carbon (3DPC)/Co_3_O_4_ composites [40]. A Co_3_O_4_–rGO composite electrode demonstrated specific capacitance 688 F g^−1^ at loading 1–2 mg cm^−2^ [34]. Gopalakrishnan et al. have shown that at a load of 1 mg cm^−2^, the typical specific capacitance is 239.5 F g^−1^ for pure Co_3_O_4_ and 395.04 F g^−1^ for Co_3_O_4_/graphite composite [54]. Kwak et al. have shown that at mass loadings over 10 mg cm^−2^, which is considered as ultrahigh loading, the specific capacitance is much lower than the theoretical one [55]. Meng et al. synthesized porous Co_3_O_4_ particles by a solid-state conversion process that has shown a specific capacity of 150 F g^−1^ at a current density of 1 A g^−1^ and a loading of 8 mg cm^−2^ after 3400 cycles [56].

By the analysis of performance metrics, Gogotsi et al. have shown [57,58] that at least 10 mg cm^−2^ loading is very important in order to predict the performance of commercial electrodes of electrochemical capacitors. It is necessary that the active layer of the electrodes be at least 100 µm thick and load at least 10 mg cm^−2^. Such high loading is difficult to achieve using the hydrothermal method or electrodeposition, although these methods provide good electrical and mechanical contact with the conductive substrate. The volume specific capacitance in the manufacture of supercapacitors is also very important; highly porous materials and aerogels do not satisfy this condition. The method of forming electrodes using active material powder and a suitable binder is adequate to achieve high loading [31,34,37,42,46,49,51]. This method is practically important due to the scaling potential; therefore, it is being intensively developed. The relatively low value of the specific capacitance of electrodes fabricated in this way is due to the fact that at a high load, a part of the active material that makes up the capacitive electrode may not have strong physical and electrical contact with the substrate. Correspondingly, the electrical properties of the layers deposited on the conductive substrate deteriorate, and only a part of the active material participates in redox reactions and the formation of the electrode capacitance. In addition, if the particles of the active material are large, the interior of the particles also does not participate in the creation of pseudo-capacity. Thus, an important issue of materials for supercapacitor electrodes is their modification to achieve high capacitance and high mass loading.

We have previously shown [59] that Co_3_O_4_ nanoparticles synthesized by aerosol pyrolysis can be modified by reductive annealing in a hydrogen atmosphere, and electrodes made from this material demonstrate a significantly higher specific capacitance than electrodes made from the original cobalt oxide. However, during recovery annealing, the undesirable sticking and coarsening of nanoparticles occurred. Therefore, a method for modifying finished electrodes needs to be developed. The binder should be used to achieve high loading, and the study of the stability of the structure and parameters of such electrodes during recovery annealing should be carried out.

In the present work, we synthesized Co_3_O_4_ nanoparticles by a simple chemical deposition method, fabricated electrodes using a PVDF binder on a nickel foam substrate, and worked out a reduction annealing procedure in a hydrogen atmosphere. The capacity of the modified electrodes increased by more than four times immediately after treatment and by another 10% when aging in air for 15 days under ambient conditions until stabilization. The effect was achieved due to the small size of the oxide particles; therefore, the complete recrystallization of the nanocrystalline structure Co_3_O_4_ → CoO occurs as a result of reductive annealing, and the reverse transition CoO → Co_3_O_4_ occurs during in situ oxidation. Such recrystallization led to crystallization purification and an increase in the concentration of oxygen vacancies; as a result, the surface was activated. The specific capacitance in the three-electrode system was 403.8 F g^−1^ at a current density of 1 A g^−1^ at low loading (1.2 mg cm^−2^) and 5.18 F cm^−2^ at a scan rate of 0.008 V s^−1^ at high loading (41 mg cm^−2^). An asymmetric capacitor with an activated carbon negative electrode and a modified cobalt oxide positive electrode demonstrated an operating potential range of 1.5 V in KOH electrolyte, the capacitance of the hybrid capacitor was 2.04 F cm^−2^ at a current density of 20 mA cm^−2^ (0.5 A g^−1^), and very high cyclic stability and a good rate capability of 34% at 10 A g^−1^ was obtained.

## 2. Materials and Methods

### 2.1. Chemicals

The reagent grade Cobalt (II) acetate tetrahydrate (C_4_H_6_CoO_4_·4H_2_O), sodium acetate trihydrate (CH_3_COONa·3H_2_O), sodium hydroxide (NaOH) and N-methyl-2-pyrrolidone (NMP) were purchased from Sigma Aldrich, St. Louis, MO, USA. The binder PVDF Kynar was purchased from PolyK, 2124 Old Gatesburg Rd, State College, PA, USA. MilliQ water (18.2 Mohm × cm) was produced in-house with a type I ultrapure water purification system from Water Purification System AQUAMAX—Ultra 370 Series (YL Instrument Co., Anyang, Korea). Activated carbon for non-Faradaic negative electrodes was purchased from Fuzhou Yihuan Carbon Co. (Fuzhou City, China).

### 2.2. Synthesis

A simple chemical precipitation method was used to synthesize cobalt oxide nanoparticles (Co_3_O_4_ NPs). Cobalt (II) acetate tetrahydrate (0.01 mol) and sodium acetate trihydrate (0.2 mol) were dissolved in 100 mL of water. In addition, 0.02 mol NaOH was dissolved in 50 mL of water. The alkali solution was added to the salt solution with vigorous stirring at room temperature. The solution was stirred for 20 min, and then, the reaction was stopped by the addition of water, bringing the volume of the solution to 1 L. The precipitations were washed with deionized water several times by centrifugation; then, the precipitate was washed to neutral pH, filtered, and stored in ethanol. For the manufacture of electrodes, the resulting brown powder was dried at 90 °C overnight. After drying, the sample was annealed at 200, 350, and 600 °C in air.

### 2.3. Preparation of Electrodes

For the preparation of electrodes, either the pristine as-synthesized powder or pre-annealed one was mixed with 8 wt % of PVDF powder, and the mixture was ground in an agate mortar. Then, the mixture was transferred into a glass test tube, 6 mL of NMP was added, and the solution was thoroughly stirred on a magnetic stirrer.

Nickel foam (NF) substrates with an area of 1 cm^2^ and a thickness of 3 mm were cleaned by boiling in acetone and immersing in 10% nitric acid for several seconds, which was followed by washing in water and drying. The pseudocapacitor electrode was prepared as follows. The required amount of solution was applied to a pre-weighed NF substrate, which was followed by drying in a vacuum oven. Low loading (≈1 mg cm^−2^) NPs–NF electrodes were prepared by applying 20 µL of NMP solution with Co_3_O_4_ NPs active material to the substrate followed by vacuum drying at 60 °C for 3 h. To obtain samples with a high load, this operation was repeated for the required number of times. Then, the obtained NPs–NF electrode was pressed, and the mass of the active material was determined from the difference between the masses of the fabricated electrode and the original substrate.

The NPs–NF electrodes were annealed in hydrogen (hereinafter referred to as “H2-treatment”) at 275 °C in a quartz tube furnace in a hydrogen flow of 2 L per hour at a heating rate of 4 deg min^−1^. After annealing, the furnace was cooled to room temperature in a hydrogen atmosphere. It was necessary to let atmospheric air in with care to prevent heating of the samples due to rapid oxidation. Then, the samples were pressed at ≈10 MPa.

To make an activated carbon electrode, AC powder was mixed with carbon black (CB) and PVDF in a weight ratio of 8:1:1 and ground in an agate mortar; then, it was mixed in NMP solution, and the obtained paste was applied to nickel foam, followed by vacuum drying, after which the AC–NF electrode was pressed.

### 2.4. Characterization

A scanning electron microscope (SEM) Quanta 200i 3D (FEI, Hillsboro, OR, USA) was used to characterize morphology. An X-ray diffractometer MiniFlex (Rigaku, Tokyo, Japan) was used to perform X-ray diffraction (XRD) analysis and identify phases in the samples. XPS spectra were collected by a NEXSA X-ray Photoelectron Spectrometer (Thermo Scientific, Waltham, MA, USA). Raman spectra were recorded using a NTEGRA Spectra (NT-MDT, Zelenograd, Russia) spectrometer with a 473 nm solid-state exciting laser.

### 2.5. Electrochemical Measurements

The electrochemical capacitive performances of samples were analyzed by a potentiostat P-40X-FRA-24M (Elins, Chernogolovka, Russia) through cyclic voltammetry (CV), galvanostatic charge/discharge (GCD), and electrochemical impedance spectroscopy (EIS) measurements both in two-electrode and three-electrode systems. In the three-electrode system, a platinum electrode was used as the counter electrode along with an Ag/AgCl electrode as the reference electrode in a conventional electrochemical cell with a 3.5 M KOH electrolyte. In the two-electrode system, a Swagelok-type cell was used to analyze the electrochemical characteristics of a hybrid capacitor using a 3.5 M KOH electrolyte with an AC–NF anode, a Co_3_O_4_ NPs–NF cathode, and a paper filter separator.

## 3. Results

**Figure 1:**
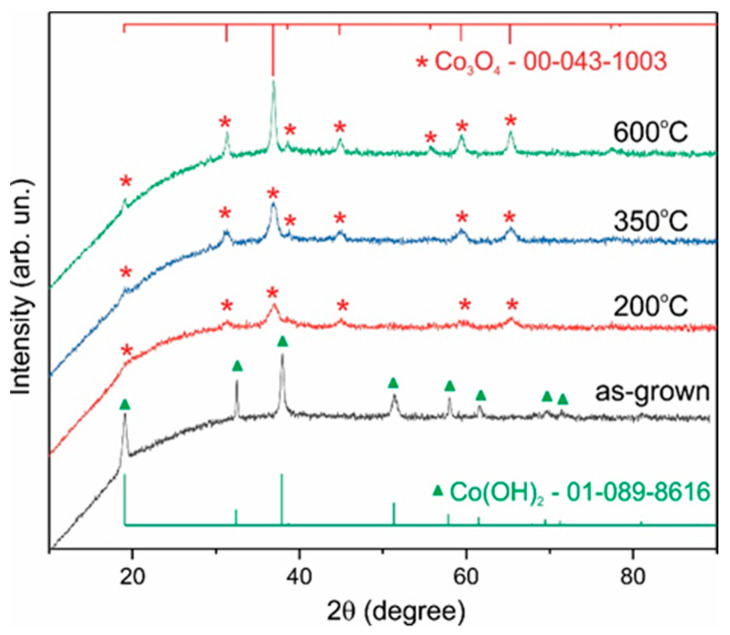
XRD patterns of the as-synthesized Co(OH)_2_ sample and Co_3_O_4_ samples after annealing in air at temperatures of 200, 350, and 600 °C.

**Figure 2:**
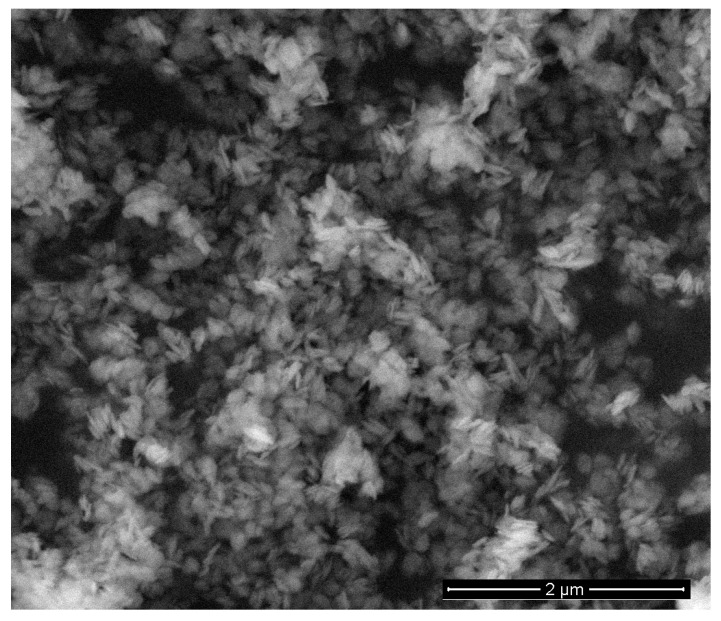
SEM images of Co_3_O_4_ powder sample annealed at 200 °C in air.

Figure 1 shows the XRD pattern of the as-synthesized sample as well as powder annealed at 200, 350, and 600 °C. The observed structures of the diffraction peaks are completely in line with the standard diffraction pattern of Co(OH)_2_ (JCPDS card No. 01-089-8616) in the as-grown sample and Co_3_O_4_ (JCPDS card No. 00-043-1003) in annealed samples. The presence of the CoO phase was not detected. The full width at half maximum (FWHM) of the peaks decreases with increasing annealing temperature. Estimation of crystallite sizes using Scherrer’s formula gives crystallite sizes of 6, 8, and 17 nm for annealing temperatures of 200, 350, and 600 °C, respectively. These nanocrystals aggregate into platelet-like porous particles (Figure S1a, Supplementary Materials), and the shape does not change after annealing at 200 °C (Figure 2). The particles are easily crushed into smaller particles after milling (Figure S1b).

**Figure 3:**
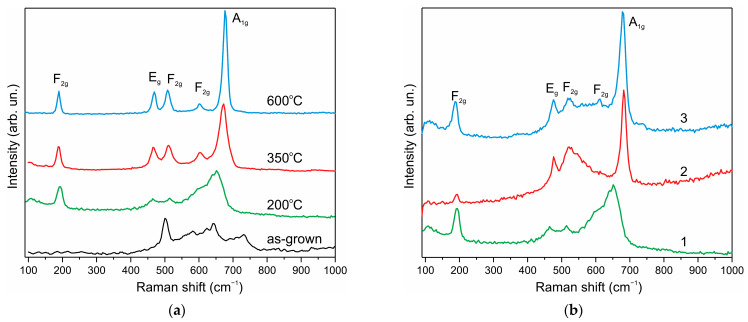
Raman spectra of Co_3_O_4_ samples: (**a**) asgrown and annealed at 200, 350, and 600 °C in the air; (**b**) after synthesis annealed at 200 °C (1), after annealing in H_2_ at 275 °C for 1 h (2), after measuring CV characteristics (3).

The Raman spectra of Co_3_O_4_ nanoparticles (Figure 3a) were measured under low excitation power of 0.035–0.122 mW. The Raman spectrum of the as-grown sample (Figure 3a, spectrum 1) can be attributed to Co(OH)_2_ nanoparticles [60]. The sample annealed at 200 °C (Figure 3a, spectrum 2) represents a weakly formed spectrum, which can be attributed to the Co_3_O_4_ spinel structure with broad lines due to the nanosize effect. Raman spectra completely corresponding to spinel Co_3_O_4_ [61,62] are observed after annealing at 350 and 600 °C.

**Figure 4:**
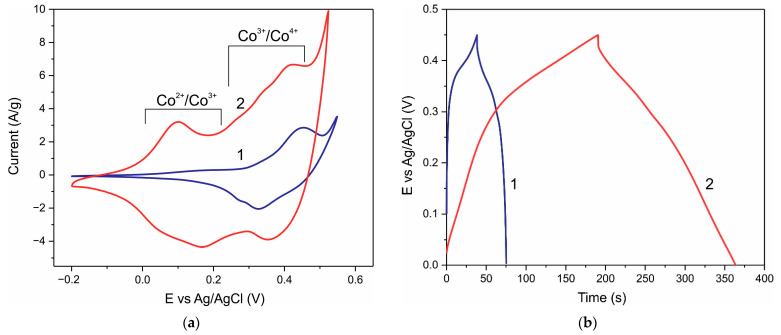
CV curves of the Co_3_O_4_ electrode with a mass loading of 1.2 mg cm^−2^ at a scan rate of 0.008 V s^−1^ (**a**); Galvanostatic charge–discharge curves at current density of 1 A g^−1^ (**b**), 1—before H2-treatment, 2—after H2-treatment. The approximate potential ranges corresponding to the Co^3+^/Co^4+^ and Co^2+^/Co^3+^ redox reactions are shown in Figure (**a**).

Cyclic voltammetry (CV) and galvanostatic charge/discharge (GCD) characteristics of a Co_3_O_4_ electrode before and after annealing in hydrogen at 275 °C for 1 h are shown in Figure 4. The CV curves of the pristine electrode show the presence of one major pair of Co^3+^/Co^4+^ redox peaks (Figure 4a, curve 1). As a consequence, the GCD curves are significantly non-linear (Figure 4b, curve 1). H2-treatment of the same electrode causes the CV curves to show significant current over a wide range of potentials (Figure 4a, curve 2); i.e., a wide range of active redox sites are created during H2-treatment.

The capacitance of the electrodes can be estimated from the CV curves using the formula:

(3) $$C_{s}=\frac{1}{2m\nu \left(V_{\max}-V_{\min}\right)}\oint^{\;}I\left(V\right)\mathrm{dV}$$

where C_s_ is the specific capacitance (F g^−1^), m is the mass of the electroactive material of the electrode (g), ν is the scan rate (V s^−1^), V_max_ − V_min_ is the potential window (V), I is the current (A), and integration is performed over one CV cycle.

The specific capacitance of the electrode with a mass loading of 1.2 mg cm^−2^ (Figure 4a) at a scan rate of 8 mV s^−1^ estimated from Formula (1) was 73.6 F g^−1^ and 324.4 F g^−1^ for the pristine electrode and the same electrode after H2-treatment, respectively; that is, the capacity increased by a factor of ≈4.4 as a result of H2-treatment. CV curves of the Co_3_O_4_ NPs electrode with a higher loading of 5.2 mg before and after H treatment at different sweep rates are shown in Figure S2.

**Figure 5:**
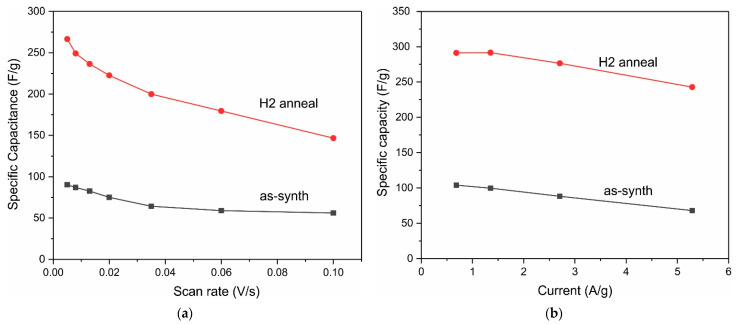
The specific capacitance of the Co_3_O_4_ NPs electrode with a loading of 5.2 mg vs. scan rate (**a**) and discharge current (**b**).

The capacity of the electrodes can also be calculated using the GCD method by the following equation:

(4) $$C=\frac{\mathrm{Idt}}{\Delta U}$$

where I is the discharge current (A), dt is the discharge time (s), C is the capacity (F), and ΔU is the voltage drop. The capacitances of the electrodes (Figure 4b) were 85.8 F g^−1^ and 403.8 F g^−1^ at a current density of 1 A g^−1^ for the pristine electrode and after H2-treatment, respectively, which is close to the CV results. As a result of H2-treatment, the capacitance increased by a factor of ≈4.7. The GCD curves (Figure 4b) before H2-treatment deviate strongly from straight lines, i.e., the Co_3_O_4_ NPs electrode is the battery type. H2-treatment leads to an increase in the range of potentials at which a significant current is observed, i.e., the spectrum of active centers involved in redox reactions expands due to Co^2+^/Co^3+^ transitions and surface states. Accordingly, the GCD curves after H2-treatment are close to straight lines; the recovered Co_3_O_4_ NPs electrode shows pseudocapacitive behavior. Figure S3 shows the GCD dependences at different discharge currents before and after H2-treatment for the Co_3_O_4_ NPs electrode with a loading of 5.2 mg, and the specific capacitance vs. scan rate and discharge current are shown in Figure 5.

CV measurements show that the capacitance of the Co_3_O_4_ electrode increases during the first 30–40 cycles (see Figure S4a Supplementary Materials). This is due to the additional oxidation of the active material, as well as an increase in the electrode surface area available to the electrolyte. However, after H2-treatment, a very significant current in the positive scan and a low current in the negative scan at the first scan are observed (see Figure S4b), which indicates in situ oxidation of the active material after H2-treatment. Up to 30–40 cycles were required to achieve equilibrium CV curves.

It should be noted that the specific capacitance of the electrodes was the same for as-grown samples (Co(OH)_2_ phase, Figure 1) and samples annealed at 200 °C (Co_3_O_4_ phase). Therefore, in situ oxidation of Co(OH)_2_ does not cause an increase in the size of nanocrystalline particles. However, the specific capacitance decreased with an increase in the annealing temperature to 350 and higher due to an increase in the crystallite size. Therefore, only as-grown samples or samples annealed at 200 °C were subsequently studied.

**Figure 6:**
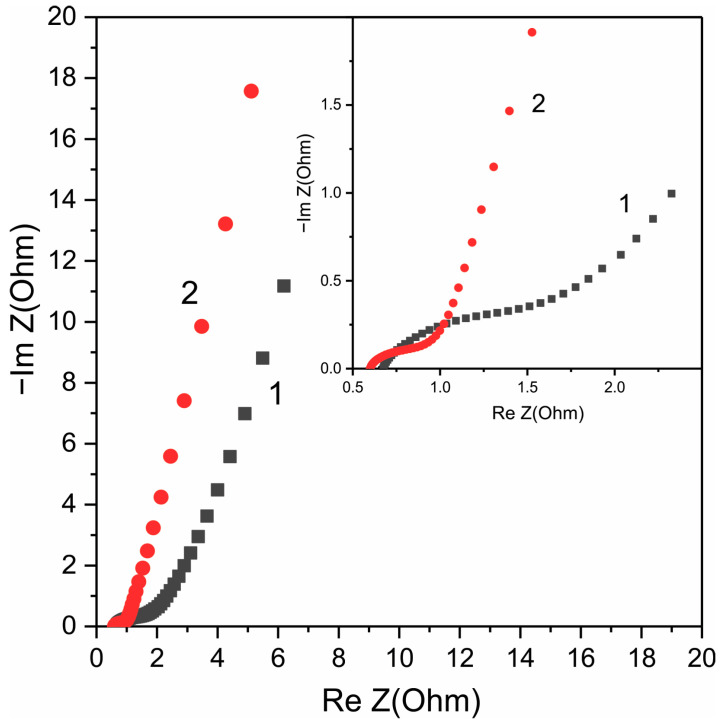
Nyquist plots of Co_3_O_4_ NPs–NF electrode with loading 3 mg before (1) and after (2) H2-treatment in the frequency range of 0.01–5 × 10^4^ Hz.

Figure 6 shows Nyquist plots of Co_3_O_4_ electrode (load 3 mg) before (1) and after (2) H2-treatment in the frequency range of 0.01–5 × 10^4^ Hz. It can be seen that the Nyquist plots consist of a high-frequency semicircle, the diameter of which depended on the potential, so this plot can correspond to the charge transfer resistance (R_ct_) or internal resistance [63]. The diameter of the semicircle at low frequencies (see the inset to Figure 5) significantly decreased after H2-treatment, which indicates an increase in the pseudocapacitive properties.

An almost straight line is observed at low frequencies; this region of the Nyquist plot corresponds to equilibrium differential capacitance, and in an ideal capacitor, it is a vertical line with a slope of 90 degrees, while a slope of 45 degrees corresponds to battery-type materials. In real supercapacitors, the slope is less than 90° due to the presence of ion diffusion in the electrodes. A significant deviation of the line in the low-frequency region from 45° and closeness to 90° (Figure 5) indicates the pseudocapacitive nature of the electrodes.

It is known that the current response i(V) depends on the potential sweep rate ν as i(V) = kν^b^, and b varies depending on the current flow mechanism, b = 0.5 if the redox reaction is controlled by semi-infinite diffusion, and b = 1 if redox reaction is surface-controlled [7,27]. The same considerations can be applied to current peaks in CV curves. It was found (Figure S5) that the b index increases after H2-treatment, which indicates a shift in the nature of the redox reaction from a diffusion to a surface mechanism. Comparison of the CV characteristics of Co_3_O_4_ electrodes before and after H2-treatment indicates that H2-treatment increases the role of subsurface active centers and causes an increase in pseudocapacitance.

**Figure 7:**
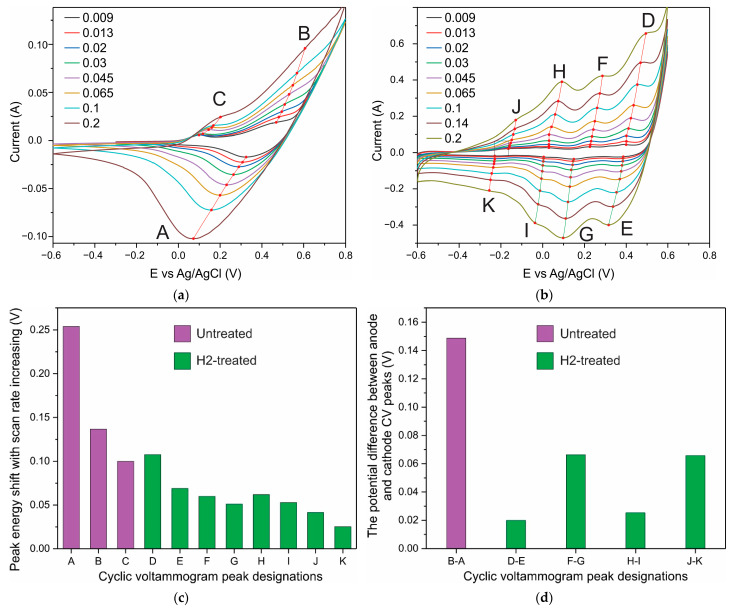
CV curves of the Co_3_O_4_ NPs–NF untreated electrode (**a**) and H2-treated one (**b**); the shifts of peak energy upon the increase in the scan rate from 0.009 to 0.2 V s^−1^ (**c**); the difference between the potential of anode and cathode peak determined at a sweep rate of 0.009 V s^−1^ (**d**).

In addition, a small potential difference or its absence between the anode and cathode current peaks at low CV sweep rates is evidence of the reversibility of the redox reaction and the capacitive nature of the system [29]. The position of the maxima of the anodic and cathodic peaks in the CV curves changes slightly with a change in the scan rate if the capacitance is controlled by surface processes. These effects are observed in H2-treated samples. The main pair of anode–cathode peaks corresponding to the Co^3+^/Co^4+^ transition is present in the initial samples (Figure 7a). H2-treated samples show several anode–cathode pairs over a wide range of potentials (Figure 7b). Figure 7c shows that as the scan rate is increased from 0.009 to 0.2 V s^−1^, the peak position shifts for the untreated sample (A–C) are significantly greater than those for the H2-treated sample (peaks D–K). In addition, the potential difference between the anodic and cathodic peaks in redox pairs in the untreated sample is large (≈150 mV), and it decreases two or more times as a result of H2-treatment (Figure 7d). Thus, near-surface centers increase the contribution to the current response as a result of H2-treatment.

In addition, the stability of the H2-treatment effect was investigated. CV and GCD measurements for the same electrode were carried out before and after H2-treatment as well as after aging under ambient conditions. Surprisingly, the capacitance increased by ≈10% within two weeks during air aging followed by stabilization (Figure S6).

Previously, reduction treatment was investigated [64], the treatment of Co_3_O_4_ nanowires in NaBH_4_ at room temperature led to a significant improvement in electrochemical performance and a threefold increase in supercapacitor capacity compared to the pristine Co_3_O_4_. At the same time, XRD data indicated that the crystal structure of Co_3_O_4_ did not change after NaBH_4_ treatment. The spectra of X-ray photoemission spectroscopy show that some of the Co^3+^ ions in the near-surface region were reduced to Co^2+^ and formed new oxygen vacancies, which, as expected, leads to the increased conductivity and high electrochemical activity of Co_3_O_4_. The mass loading of electrodes was about 0.2 mg cm^−2^.

We also carried out a reduction treatment of our Co_3_O_4_ NPs–NF electrodes with NaBH_4_ at room temperature. After treating the electrode in a fresh 2 M NaBH_4_ solution at room temperature for 2 h, the capacitance determined from the CV data at a scan rate of 0.005 V s^−1^ increased from 128.8 F g^−1^ to 228 F g^−1^, and the GCD data showed an increase in capacitance from 160.8 to 304.8 F g^−1^ at 1.4 A g^−1^, while the shape of the CV curves remained almost unchanged (Figure S7a), and the GCD curves remained far from straight lines (Figure S7b). Therefore, the effect of treatment in NaBH_4_ solution is significantly different from that of H2-treatment.

To clarify the nature of the enhancement of the pseudocapacitive properties of Co_3_O_4_ upon H2-treatment, Raman spectra and XRD data of NPs–NF electrodes subjected to H2-treatment were measured. Figure 3b shows the Raman spectra of the Co_3_O_4_ electrode on nickel foam before (spectrum 1) and after H2-treatment at 275 °C for 1 h (spectrum 2) and after measuring the CV curves (spectrum 3). The spectrum of the pristine electrode is a weakly formed spectrum, which can be attributed to the structure of the Co_3_O_4_ spinel.

H2-treatment modifies the Raman spectrum (Figure 3b, curve 2), which can be attributed to a mixture of spinel Co_3_O_4_ and cubic CoO phases, since the broad line peaking at 525 cm^−1^ clearly indicates the presence of the cubic CoO phase [61,62]. This CoO phase is metastable, since at high laser excitation power (0.9 mW), the CoO spectrum (Figure 3b) is modified into the Co_3_O_4_ spectrum due to oxidation in air. Interestingly, after measuring the CV curves, the contribution of cubic CoO disappears from the Raman spectra, and the spectrum (curve 3) becomes closer to Co_3_O_4_ (can be compared with the spectrum 350 °C in Figure 3a) than the original spectrum 1 in Figure 3b. This proves that in situ oxidation of CoO → Co_3_O_4_ takes place during CV measurements similar to oxidation in the air under high-power laser excitation.

**Figure 8:**
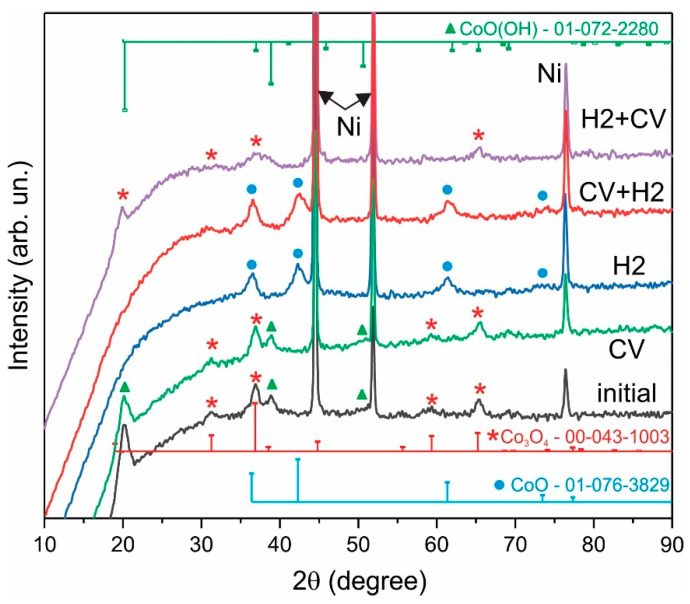
XRD patterns of the Co_3_O_4_ NPs–NF electrode after annealing in air at 200 °C (initial), in situ oxidation (CV), H2-treatment (H2), in situ oxidation followed by H2-treatment (CV + H2), and H2-treatment followed by in situ oxidation (H2 + CV).

**Figure 9:**
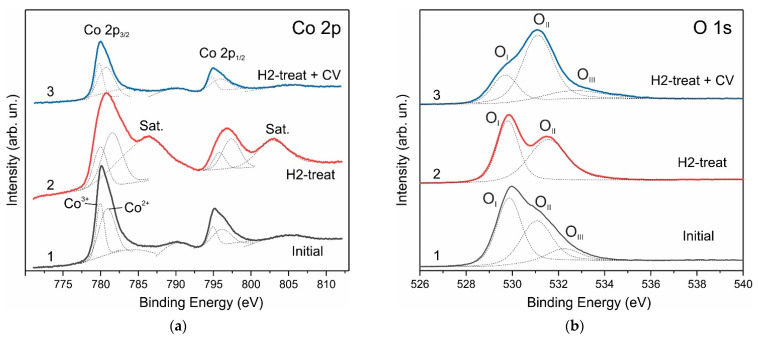
High-resolution Co 2p (**a**) and O 1s (**b**) XPS spectra for the Co_3_O_4_ NPs–NF electrode: 1—spectrum of pristine electrode before H2-treatment; 2—spectrum after H2-treatment, and 3—after H2-treatment followed by CV measurement. The dotted lines demonstrate the decomposition of the spectra into individual components.

H2-treatment also leads to a significant change in XRD patterns (Figure 8). The initial sample is dominated by the cubic phase of spinel Co_3_O_4_ (PDF Card No.: 00-043-1003), and in situ oxidation does not change the phase composition. After H2-treatment, a cubic rock salt structure CoO is formed (PDF Card No.: 01-076-3829). The surface of nanocrystals of this phase is prone to oxidation; therefore, after CV measurements, the in situ oxidation of the CoO phase to the Co_3_O_4_ phase occurs. The XRD pattern H2+CV (Figure 9) again demonstrates the presence of a spinel structure of Co_3_O_4_ nanocrystals, while the intensity of the Co_3_O_4_ reflections decreases and the half-width increases compared to the initial XRD pattern.

XPS Co 2p spectra confirm the effect of the structural transformation of Co_3_O_4_ nanocrystals as a result of H2-treatment of electrodes and in situ oxidation. Figure 9 shows high-resolution Co 2p XPS spectra for the Co_3_O_4_ NPs–NF electrode. The peaks in the XPS spectrum of the pristine electrode are at energies of 780.1 eV (Co 2p_3/2_) and 795.0 eV (Co 2p_1/2_), and the intensities of the higher energy satellite peaks are low, which fully corresponds to the standard XPS spectrum of Co_3_O_4_ [65].

H2-treatment results in broadening the Co 2p_3/2_ and Co 2p_1/2_ peaks of the Co 2p spectra and increasing to energies of 780.7 eV and 796.7 eV, correspondingly; also, intense satellites at energies of 786.5 eV and 803.0 eV appear, which indicates the presence of Co^2+^. The Co 2p XPS spectrum (curve 2) completely coincides with the standard spectrum of CoO [65]. The XPS spectrum of electrodes subjected to in situ oxidation decreases in intensity but completely repeats the shape of the XPS spectrum of pristine Co_3_O_4_ electrode.

The O 1s spectra of the pristine Co_3_O_4_ NPs–NF electrodes (Figure 9b) are deconvoluted into three peaks, a low binding energy peak O_I_ at 530 eV, a middle binding energy peak O_II_ at 531.2 eV and a high binding energy peak O_III_ at 532.4 eV. The H2-treated electrode demonstrates only two O 1s XPS line O_I_ at 530 eV and O_II_ at 531.6 eV, and again, a three O 1s lines spectrum is observed in the in situ oxidized electrode at 529.5 eV, 531.2 eV and 532.5 eV. The line O_I_ at 529.5 eV is attributed to lattice oxygen, the high binding energy peak O_III_ can be attributed to adsorbed oxygen, and the O_II_ peak at 531.2 eV can be attributed to oxygen defect with a coordination number less than four, i.e., to an oxygen vacancy. The area of the O_II_ peak with respect to the O_I_ peak increases after H2-treatment, and the O_II_ peak becomes the main one in the in situ oxidized sample. According to the Raman and XRD results, the main phase in this sample is Co_3_O_4_. This is strong evidence that the concentration of oxygen vacancies in the H-treated sample is significantly higher than in the pristine one.

Therefore, joint consideration of the XRD, Raman, and XPS measurement results allows us to conclude that both the surface and the volume of nanoparticles undergo a transformation Co_3_O_4_ → CoO → Co_3_O_4_ as a result of H2-treatment and subsequent in situ oxidation. Such recrystallization of the Co_3_O_4_ phase in CoO during annealing in a hydrogen atmosphere can cause surface cleaning and the activation of surface centers. An increase in the concentration of oxygen vacancies in Co_3_O_4_ during in situ oxidation CoO → Co_3_O_4_ will lead to an increase in the electrochemical properties of the electrode.

**Figure 10:**
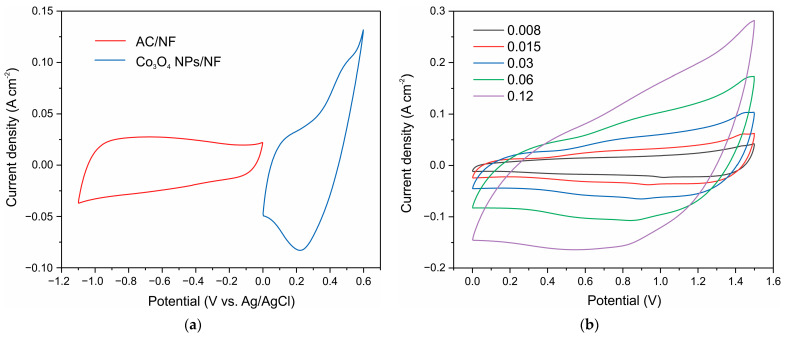

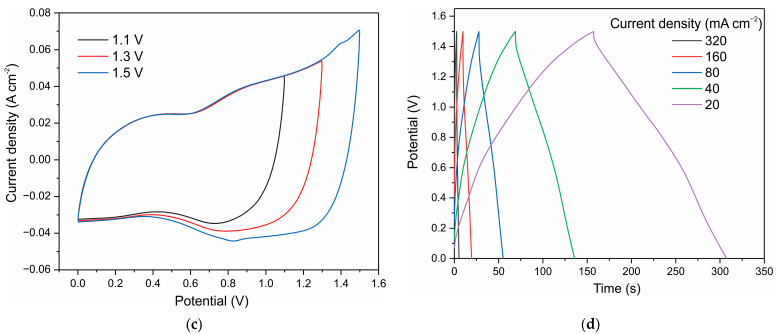
CV curves at 0.008 V s^−1^ of the positive (reduced Co_3_O_4_ NPs–NF) and negative (AC–NF) electrodes (**a**); CV curves of Co_3_O_4_ NPs–AC capacitor at different scan rates of 8–60 mV s^−1^ (**b**); CV curves of the capacitor at different voltage windows, and 8 mV s^−1^ scan rate (**c**); and GCD curves of the capacitor at different current densities (**d**).

To evaluate the supercapacitor application of the materials, the electrochemical properties of Co_3_O_4_ nanoparticles were evaluated in a two-electrode system. A positive electrode was fabricated with a high loading of 41 mg Co_3_O_4_ nanoparticles on 1 cm^2^ nickel foam; this electrode exhibited a capacitance of 5.18 F as estimated from the CV curves at 8 mV s^−1^ in the 0–0.6 V range (Figure 10a). An AC–NF electrode on nickel foam with an area of 1 cm^2^ was prepared as a negative electrode; its capacitance from CV curves at a rate of 8 mV s^−1^ was 2.65 F (Figure 10a). Using a paper filter as a separator and a 3.5 M KOH electrolyte, a capacitor was assembled, whose CV curves at different scan rates of 8–60 mV s^−1^ are shown in (Figure 10b). The figure shows that pronounced redox pairs characteristic of battery-type materials are absent.

The capacitance of the resulting capacitor estimated from the CV curves at 8 mV s^−1^ was 2.08 F cm^−2^. The capacitor shows good electrochemical behavior and reversible reactions in a potential window of 1.5 V (Figure 10c).

The GCD curves (Figure 10d) ensure that the stable operating potential of the resulting capacitor is 1.5 V, the charge–discharge curves are close to straight lines, the capacitance is 2.04 F cm^−2^ and the Coulomb efficiency is 95.5% at a current of 20 mA cm^−1^ and increasing up to 99.5% with increasing current. Figure S8 shows Nyquist plots of Co_3_O_4_ NPs–AC structures in the frequency range of 0.01–5 × 10^4^ Hz at a bias voltage of 0.5 V and 1 V. It can be seen that the Nyquist plots have the form typical of supercapacitors, and the charge transfer resistance (or internal resistance [63]) is small: about 0.75 ohms.

Figure S9 shows the charge/discharge cyclic stability of the 1 cm^2^ supercapacitor under the current of 100 mA cm^−2^. As can be seen, the capacitance of the Co_3_O_4_ NPs–AC–NF capacitor increases during the first 2000 cycles and remains stable up to 5000 cycles.

## 4. Conclusions

Co_3_O_4_ nanoparticles about 6 nm in size were synthesized by a simple chemical deposition method, which is often used as a standard method for creating materials for the positive electrodes of hybrid supercapacitors. The recovery treatment method was used to modify the properties of electrodes consisting of recovery nanoparticles deposited on a nickel foam substrate using an NMP binder and without using a carbon filler. It is shown that an effective improvement in the electrochemical activity of electrodes made of reduced nanoparticles is achieved by heat treatment in a hydrogen atmosphere at a temperature of 275 °C for 1 h.

This H2-treatment increases the specific capacitance by more than four times, does not destroy the electrode structure, and the binder does not degrade. The recovery treatment leads to the activation of the surface pseudocapacitance, and the surface-controlled redox reaction makes the main contribution to the current response. The small potential difference between the anode and cathode peaks of the CV curves and the weak dependence of the peak positions on the scanning rate indicate that surface processes make the main contribution to the capacity. The wide potential window at which an intense current response is observed, as well as the close-to-rectilinear shape of the discharge curves, indicates that the recovery treatment activates a wide range of redox reactions, and, along with the Co^3+^/Co^4+^ pair, the Co^3+^/Co^4+^ reaction makes a significant contribution to the current response.

The results of Raman, XRD, and XPS studies of Co_3_O_4_ electrodes show that the H2-treatment results in the transformation of the structure of Co3O4 nanocrystals into the CoO phase. The CoO phase is unstable during electrochemical measurements of CV curves, and a reverse transition from the CoO to Co_3_O_4_ structure takes place during in situ oxidation. Such recrystallization leads to activation of the surface of nanoparticles and creates oxygen vacancies; the concentration in the H2-treated sample is much higher than in the initial sample, as evidenced by XPS spectra; and the electrochemical activity of the surface is enhanced. The storage of electrodes in ambient conditions does not cause degradation; on the contrary, the specific capacitance increased by 10% within 15 days.

A hybrid capacitor, with a Co_3_O_4_ positive electrode, a high loading of 41 mg Co_3_O_4_ nanoparticles on a 1 cm^2^ nickel foam, and an activated carbon negative electrode showed capacitance of 2.08 F cm^−2^ at a CV scan rate of 8 mV s^−1^ and 2.04 F cm^−2^ at 20 mA cm^−2^ and high cyclic stability (5000 cycles).

For the practical application of modified Co_3_O_4_ pseudocapacitive electrodes, it is necessary to develop a composite based on a highly conductive matrix (for example, carbon) to further increase the mass loading and lower the series resistance, which limits the power of the capacitor.

## Data Availability

The data presented in this study are available on request from the corresponding author.

## Supplementary Materials

The following supporting information can be downloaded at: https://www.mdpi.com/article/10.3390/nano12203669/s1, Figure S1: SEM images of Co_3_O_4_ after synthesis (a) and after annealing in air and milling (b); Figure S2: CV curves of the same Co_3_O_4_ NPs–NF electrode weighing 5.1 mg before and after H-treatment at different sweep rates; Figure S3. GCD dependences at different discharge currents (a—0.69 A/g, b—1.35 A/g, c—2.71 A/g, and d—5.29 A/g) before and after H2-treatment for Co_3_O_4_ NPs-NF electrode weighing 5.2 mg; Figure S4: Changes in CV curves for Co_3_O_4_ electrodes during the first 33 cycles. The curve numbers correspond to the cycle number. CV curves are shown for the electrode before H2-treatment (a) and after H2-treatment (b); Figure S5: The peak current intensities vs. the sweep rate ν in the pristine electrode (a) and in the H2-treated electrode. The insets show the CV curves and indicate the corresponding CV peaks; Figure S6: CV and GCD curves for the same Co_3_O_4_ electrode before (1) and after H2-treatment (2), as well as after aging for 2 weeks under ambient conditions (3). Sample weight 10.5 mg, (a) CV rate 10 mV s^−1^, (b) GCD current 1.2 A g^−1^; Figure S7: CV and GCD curves for the same Co_3_O_4_ electrode before (1) and after treatment (2) in NaBH_4_. The weight of the electrode is 3 mg, CV scan rate of 5 mV s^−1^, GCD current of 1.42 A g^−1^; Figure S8: Nyquist plots of Co_3_O_4_ NPs–AC capacitor in the frequency range of 0.01–5×10^4^ Hz at bias voltage of 0.5 V (1) and 1 V (2); Figure S9: Cyclic stability of the capacitance and Coulomb efficiency of the Co_3_O_4_ NPs–AC–NF capacitor with an area of 1 cm^2^ under a current of 100 mA cm^−2^ (a); GCD curves of the 1000th, 2000th, and 5000th cycles (b).
