# Supplementary material for: Efficient Recovery Annealing of the Pseudocapacitive Electrode with a High Loading of Cobalt Oxide Nanoparticles for Hybrid Supercapacitor Applications
Source: Nanomaterials (Basel). 2022 Oct 19;12(20):3669. doi: 10.3390/nano12203669 (PMC9610740; doi:10.3390/nano12203669)
Supplement: Supplementary file 1 [file nanomaterials-12-03669-s001.zip › nanomaterials-1966946-supplementary.pdf]

nanomaterials

MDPI

Supplementary Material

Efficient Recovery Annealing of the Pseudocapacitive Electrode with a High Loading of Cobalt Oxide Nanoparticles for Hybrid Supercapacitor Applications

Khabibulla A. Abdullin [1,2,*], Maratbek T. Gabdullin [3], Zhanar K. Kalkozova [1,2], Shyryn T. Nurbolat [1,2] and Mojtaba Mirzaeian [4]

[1] National Nanotechnology Laboratory of Open Type (NNLOT), Al-Farabi Kazakh National University, Al-Farabi Avenue 71, Almaty 050012, Kazakhstan
[2] Institute of Applied Science & Information Technology, Shashkin Str. 40–48, Almaty 050040, Kazakhstan
[3] Research Center of Renewable Energy and Nanotechnology, Kazakh-British Technical University, Tole bi st. 59, Almaty 050000, Kazakhstan
[4] School of Computing, Engineering and Physical Sciences, University of the West of Scotland, Paisley PA1 2BE, UK
* Correspondence: kh.abdullin@physics.kz

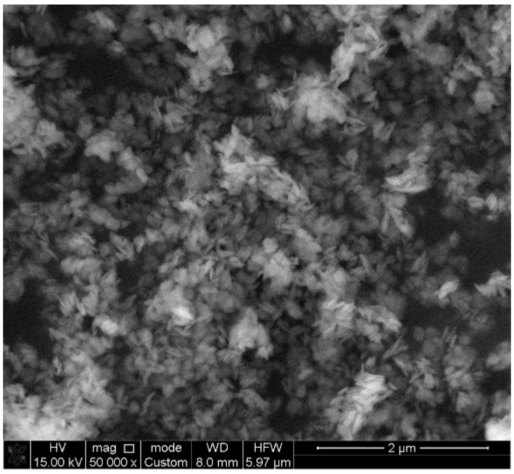

a

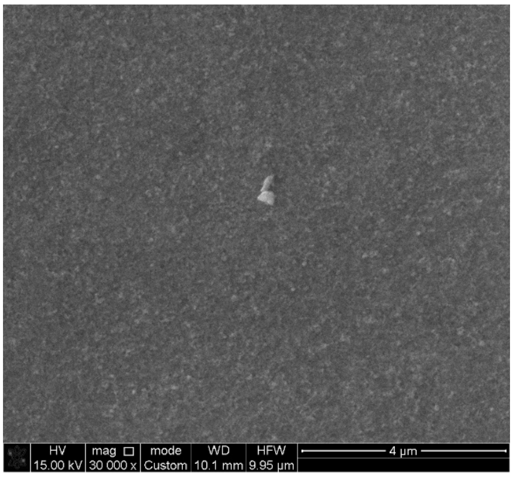

b

Figure S1. SEM images of Co_3O_4 after synthesis (**a**) and after annealing in air and milling (**b**).

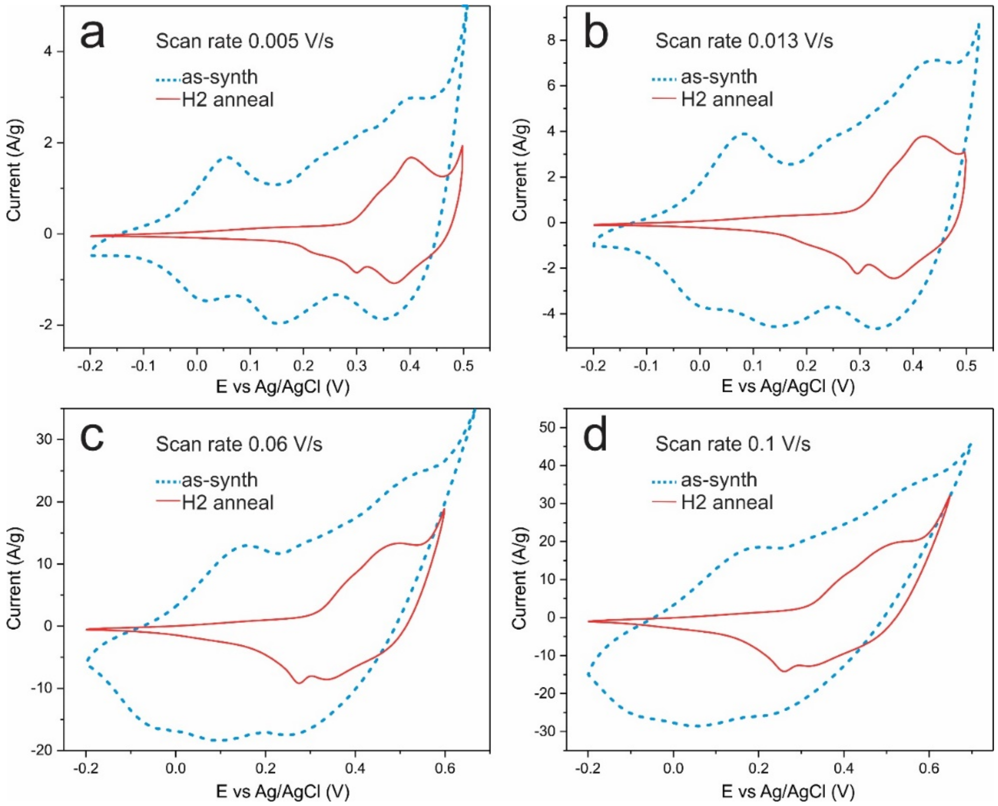

Figure S2. CV curves of the same Co_3O_4 NPs-NF electrode weighing 5.1 mg before (dashed lines) and after H-treatment (solid lines) at different scan rates: 0.005 V s^{-1} (**a**); 0.013 V s^{-1} (**b**); 0.06 V s^{-1} (**c**); and 0.1 V s^{-1} (**d**).

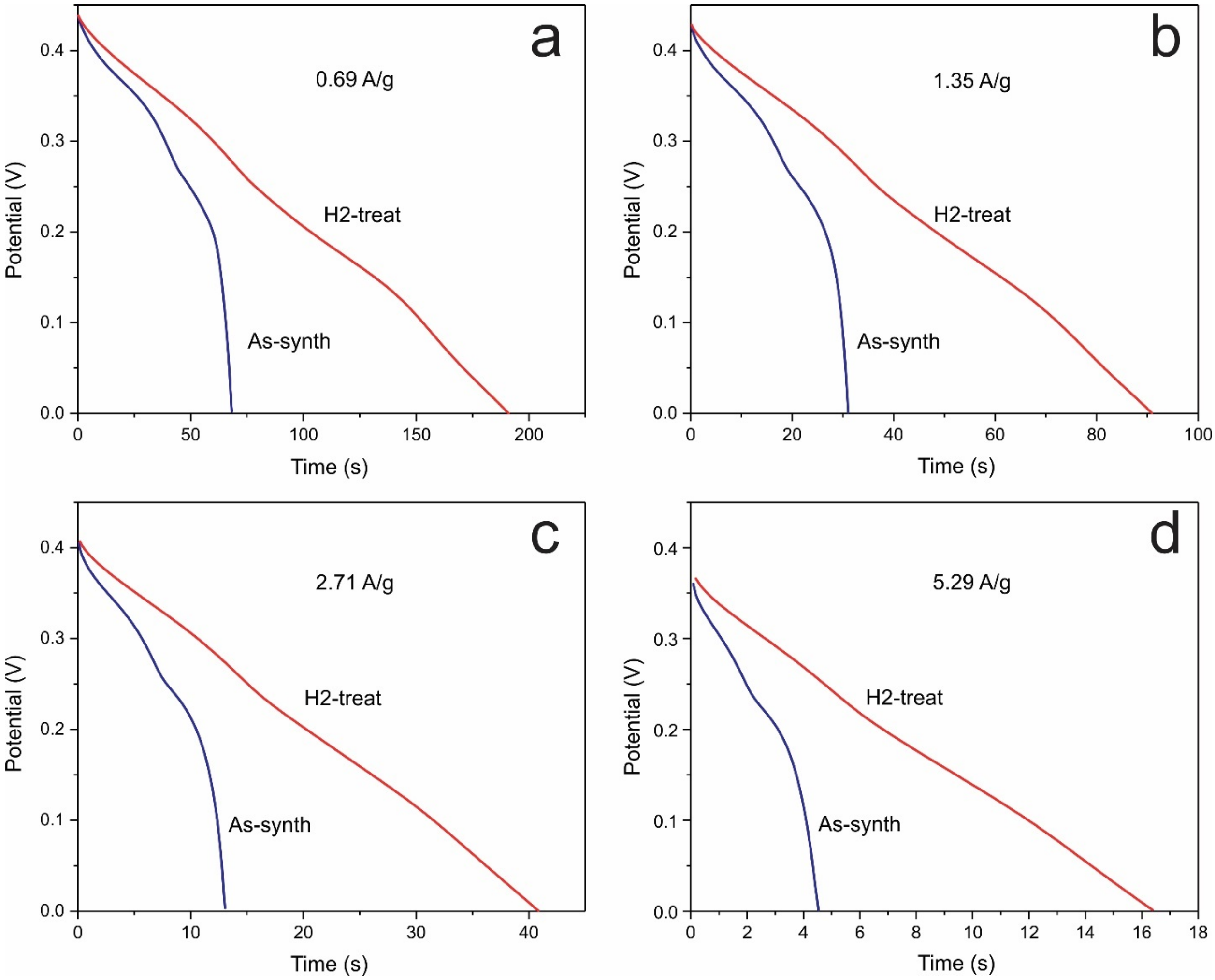

Figure S3. GCD dependences at different discharge currents (**a** – 0.69 A/g, **b** – 1.35 A/g, **c** – 2.71 A/g, and **d** – 5.29 A/g) before (black curves) and after H2-treatment (red curves) for Co_3O_4 NPs-NF electrode weighing 5.2 mg.

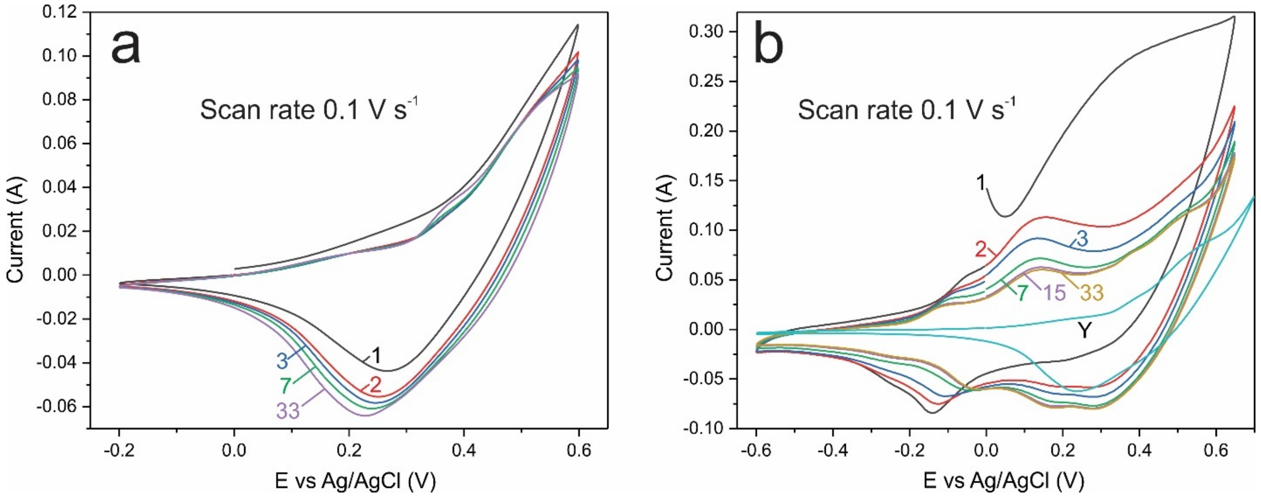

Figure S4. Changes in CV curves for Co_3O_4 electrodes during the first 33 cycles. The curve numbers correspond to the cycle number. CV curves are shown for the electrode before H2-treatment (**a**) and after H2-treatment (**b**).

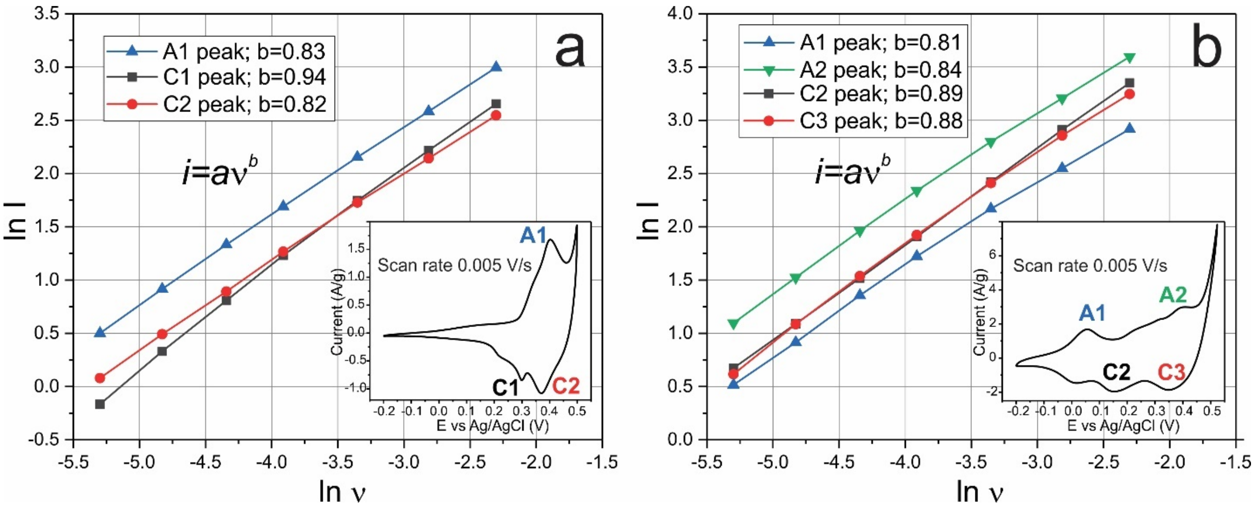

Figure S5. The peak current intensities vs. the sweep rate ν in the pristine electrode (**a**) and in the H2-treated electrode (**b**). The insets show the CV curves and indicate the corresponding CV peaks.

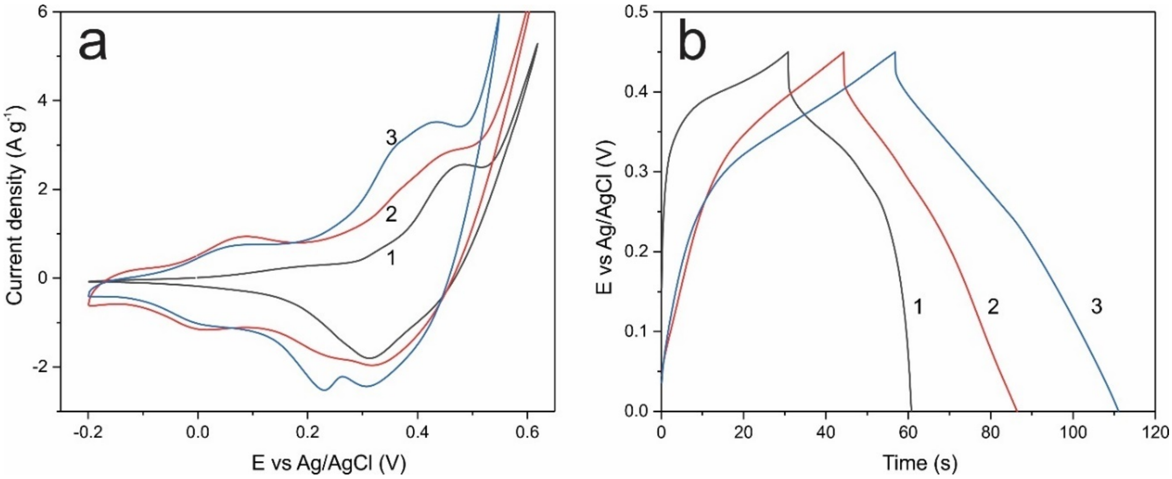

Figure S6. CV and GCD curves for the same Co_3O_4 electrode before (1) and after H2-treatment (2), as well as after aging for 2 weeks under ambient conditions (3). Sample weight 10.5 mg, (**a**) CV rate 10 mV s^{-1}, (**b**) GCD current 1.2 A g^{-1}.

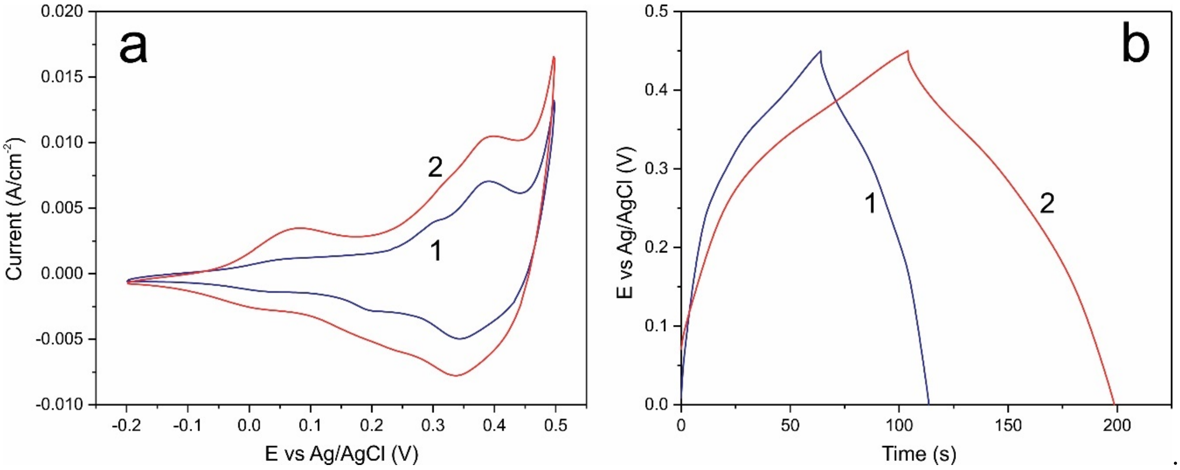

Figure S7. CV (**a**) and GCD (**b**) curves for the same Co_3O_4 electrode before (1) and after treatment (2) in BH_4Na. The weight of the electrode is 3 mg, CV scan rate of 5 mV s^{-1}, GCD current of 1.42 A g^{-1}.

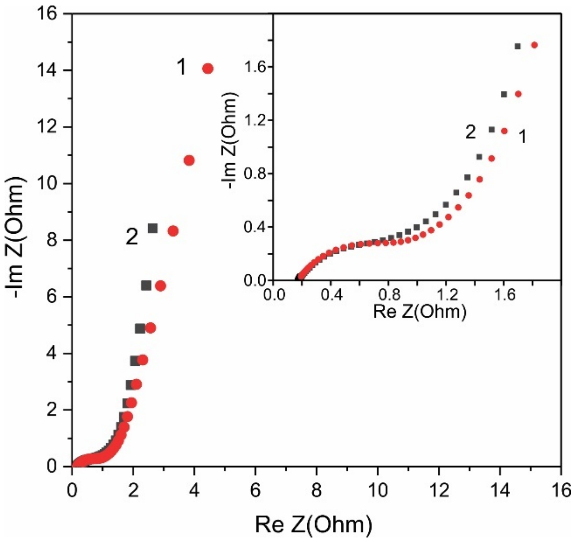

Figure S8. Nyquist plots of Co_3O_4 NPs-AC structure in the frequency range of 0.01–5×10^4 Hz at bias voltage of 0.5 V (1) and 1 V (2).

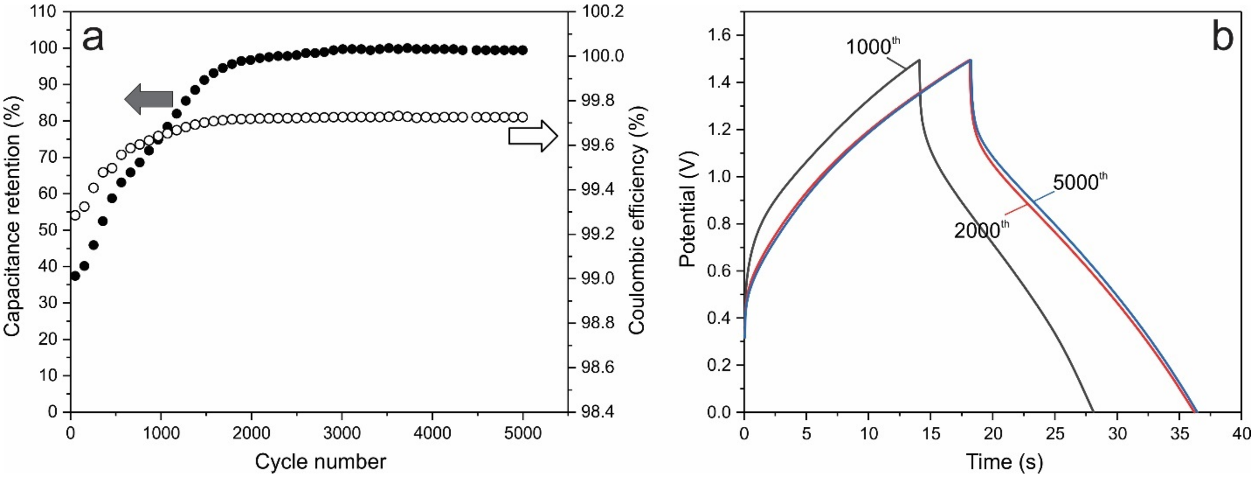

Figure S9. Cyclic stability of the capacitance and Coulomb efficiency of the Co_3O_4 NPs–AC-NF capacitor with an area of 1 cm^2 under a current of 100 mA cm^{-2} (**a**); GCD curves of the 1000th, 2000th, and 5000th cycles (**b**).
